# Evolution of the Chronic Venous Leg Ulcer Microenvironment and Its Impact on Medical Devices and Wound Care Therapies

**DOI:** 10.3390/jcm12175605

**Published:** 2023-08-28

**Authors:** Gisele Abreu Coelho, Philippe-Henri Secretan, Lionel Tortolano, Loïc Charvet, Najet Yagoubi

**Affiliations:** 1 Laboratoire Matériaux et Santé, Université Paris-Saclay, 91400 Orsay, France; philippe-henri.secretan@universite-paris-saclay.fr (P.-H.S.); lionel.tortolano@universite-paris-saclay.fr (L.T.); najet.yagoubi@universite-paris-saclay.fr (N.Y.); 2 Urgo Recherche Innovation et Développement, 21300 Chenove, France; l.charvet@fr.urgo.com; 3 Department of Pharmacy, Assistance Publique-Hôpitaux de Paris, Groupe Hospitalier Henri Mondor, 94000 Créteil, France

**Keywords:** venous leg ulcer, wound progression, wound healing, wound infection, wound dressings, simulated wound fluid

## Abstract

Studies have reported that the constituents of the wound microenvironment are likely to have critical roles in the degradation and fate of the polymeric matrix and the compounds dissolved in the wound dressing matrix. Thus, chronic wound assessment and the design of effective medical devices and drug products for wound care partly rely on an in-depth understanding of the wound microenvironment. The main aim of this review is to identify and discuss the different stages of chronic wound progression, focusing on the changes in the biochemical composition of the wound microenvironment, with particular attention given to venous leg ulcers (VLUs), as they are one of the most prevalent chronic wound aetiologies. The pathophysiology of venous ulcers is detailed, followed by a thorough review of what is known about the VLU microenvironment and its changes as a function of the evolution of the VLU. Simulating conditions for VLU are then discussed with the view of highlighting potentially relevant simulating media as a function of VLU evolution for a better assessment of biological safety, in particular medical devices intended to be in contact with these wounds.

## 1. Introduction

Chronic wounds are a prevalent medical condition that can significantly impact the lives of those affected. This condition causes functional limitations and emotional distress, leading to decreased quality of life for patients. Additionally, the treatment is prolonged, costly for health systems, and has a significant social impact as it contributes to work absence. The problem of chronic wounds is increasing worldwide with an ageing population and a rise in risk factors such as smoking, obesity, and diabetes. The prevalence of chronic wounds of mixed aetiologies is estimated at 2.21 per 1000 people, with chronic leg ulcers (CLUs) being evoked in most epidemiological studies [1]. CLU affects 0.6–3% of people over 60 years, increasing to over 5% for people over 80 years, and venous leg ulcers (VLUs) are the most common type of CLU, representing nearly 70% of all cases [2,3].

In that sense, designing effective medical devices and drug products for wound care is still an intense area of research. For instance, interactive, bioactive, and smart wound dressings are currently being investigated to treat chronic wounds of different aetiologies [4]. Either to improve existing wound dressings or to design new ones, understanding the wound microenvironment is crucial for effective management and treatment strategies and anticipating the behaviour of wound dressing materials when they are in contact with the exudates [5,6]. 

Indeed, studies have reported that the constituents of a wound microenvironment are likely to be key players in the degradation and fate of the polymeric matrix [7] and the compounds [8] dissolved in the wound dressing matrix. However, few studies have analyzed the chronic wound microenvironment concerning its chemical and biochemical composition in a more comprehensive way [5], especially regarding the changes in function that may exist at different stages of a chronic wound.

Changes observed in the wound surface reflect the cellular, molecular, and biochemical processes in the underlying tissue [9]. Great efforts have been made to recognize biomolecular pathways and biomarkers of healing and non-healing processes [10]. However, there are still many challenges to overcome regarding some critical aspects of wound healing, including the issue of bacterial presence and colonization in chronic wounds, which further complicates the healing process [11].

The primary objective of this review is to highlight the biochemical differences that occur during various stages of chronic wound progression, with a specific focus on elucidating these changes. Special attention will be directed towards venous leg ulcers (VLUs), one of the most common types of chronic wounds. Additionally, a secondary objective was to gather examples of the impacts of these biochemical modifications on the wound microenvironment and wound dressings. The review also gathers some information regarding simulated wound fluids used for wound care product assessment, including a comparison with the composition of wound exudates.

## 2. Pathophysiology of Venous Leg Ulcers

### 2.1. Etiologic and Pathophysiologic Classification

VLUs have a complex, multifactorial pathophysiology with a persistent inflammatory state that contributes to the apparition of ulcers and delays wound healing. Active VLU is the most severe manifestation of chronic venous disease (CVD), which is the underlying pathology leading to the apparition of VLU [12].

The Society for Vascular Surgery (SVS) recommends that all patients with venous leg ulcers be classified based on the CEAP (Clinical class, Etiology, Anatomy, Pathophysiology) classification, which is an internationally accepted standard for describing patients with all stages of CVD and has been used for reporting clinical research findings in scientific journals [12,13] (Table 1).

From an etiological point of view, CVD can result from congenital, primary, or secondary disorders. Several predisposing factors have been highlighted to promote venous hypertension, including advanced age, female sex, genetic predisposition, family history, pregnancy, estrogen levels, obesity, prolonged standing and sitting, and environmental/occupational factors [14].

### 2.2. Pathophysiological Mechanisms in CVD-VLU

VLU results from an intricate series of pathological events involving hemodynamic, cellular, and biochemical changes of both the macro and micro components of the venous system, which are eventually transmitted to the skin. In this complex scenario, the typical finding is increased ambulatory venous pressure [9].

**Figure 1:**
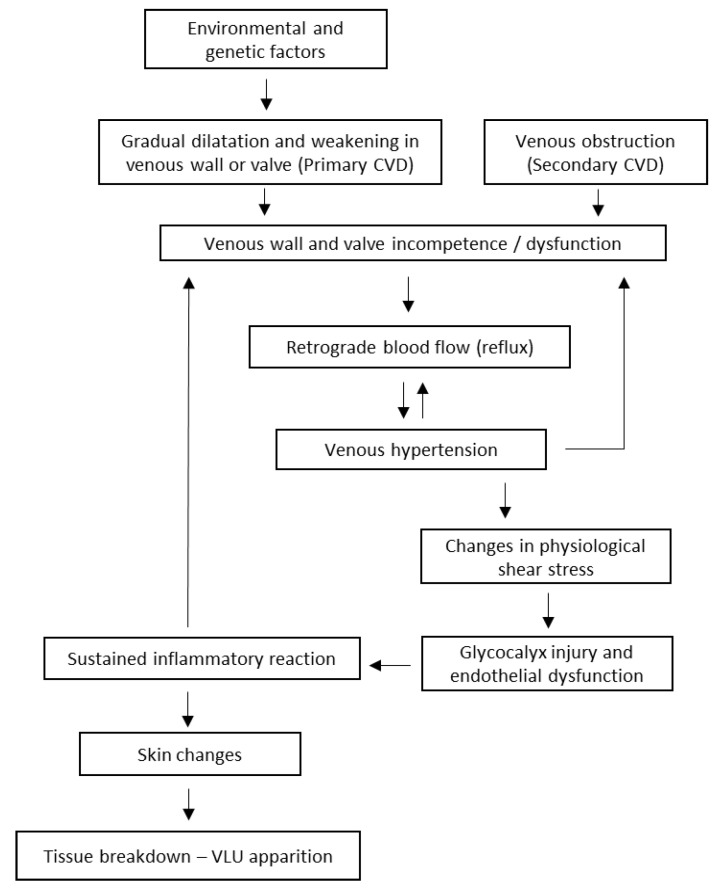
Sequence of events from CVD to VLU apparition.

Venous hypertension occurs when the pressure in the veins of the lower extremities increases due to impaired venous return. This increased venous pressure can be caused by valve dysfunction, venous obstruction, or calf muscle dysfunction. Reflux, or retrograde blood flow, is caused by venous valve incompetence and is common in patients with VLU. Valve dysfunction can lead to the pooling of venous blood, which increases venous pressure on the vein walls and alters the physiological shear stress that normally maintains blood fluidity and inhibits blood cell attachment. These mechanical stress forces can cause damage to the endothelium cells by disrupting the protective glycocalyx layer, promoting their activation, and enhancing their permeability (Figure 1) [9,14]. 

Activating endothelial cells leads to the expression of adhesion molecules such as ICAM-1, VCAM-1, and E-selectins, as well as the release of chemoattractant molecules that attract and recruit white blood cells to attach and migrate within the vein wall and surrounding tissue. Once activated, leukocytes and endothelial cells release various inflammatory and proteolytic mediators, growth factors, and chemotactic signals, which work together to target fibroblasts, vascular smooth muscle cells (VSMCs), and the extracellular matrix [9,14].

The augmented permeability of endothelial cells also causes the escape of red blood cells, which releases hemoglobin and ferric iron in the interstitial fluid. This exacerbates oxidative stress and inflammation of the adjacent tissues, leading to skin changes and perpetuating the progression of CVD [9,15].

Skin changes include eczema and pigmentation (stage C4a), which appear to be caused mainly by a hemosiderin deposit occurring in the early stages of CVD, lipodermatosclerosis (stage C4b), and a specific chronic fibrosing panniculitis (inflammation of subcutaneous fat) related to CVI [9]. Tissue breakdown leading to VLU apparition occurs as a consequence of lipodermatosclerosis progression [16]. The mechanisms described above contribute to the delayed healing of VLU by creating an unfavourable wound microenvironment since its onset (Figure 1).

## 3. Overview of the VLU Microenvironment

The chronic VLU wound bed constitutes a complex and dynamic microenvironment. In this section, we will describe the macroscopic components that characterize a chronic VLU, including exudate, granulation tissue, epithelial tissue, and sloughed or devitalized tissue. All these components may coexist in the same wound bed, regardless of the state of cicatrization. The biochemical composition of the wound bed will be depicted, and the main classes of compounds and their functions will be highlighted (see Section 3.2). A chapter on the influence of pH within the wound microenvironment is also proposed (see Section 3.3.1).

### 3.1. Structural Components of a Chronic VLU Wound Bed

#### 3.1.1. Exudate

##### Importance and Origin of Wound Exudate

The idea that a wound needed to be kept dry for proper healing was once prevalent, with exudates considered detrimental to the healing process. However, in the 1960s, research conducted by Winter [17] demonstrated that maintaining a moist environment promotes faster healing rates. This discovery revolutionized wound care practices and the development of dressing materials.

Exudate is important in the wound healing process to maintain a moist environment and provide water, electrolytes, nutrients, inflammatory mediators, white cells, and protein-digesting enzymes needed to heal [18]. However, the amount of exudate should be equilibrated; large amounts of exudate impair healing by macerating the surrounding skin and promoting infection [19,20].

Exudate is produced in the inflammatory step of wound healing (see Section 4.1). The inflammatory response involves the release of various mediators, such as histamine, which causes an increase in capillary permeability, allowing white blood cells to move out of the bloodstream and into the affected area. This process also causes the blood vessels to leak more fluid, resulting in excess fluid entering the wound site, which ultimately forms the basis of exudate [18].

##### Exudate Types

Exudate characteristics change with the function of the wound’s pathophysiologic context. The types of exudates include serous, sanguineous, serosanguineous, and purulent. Table 2 shows the main characteristics of each exudate type. The biochemistry of chronic VLU exudate is discussed in the next chapter (see Section 3.3) wound exudate composition).

#### 3.1.2. Granulation and Epithelial Tissues

Granulation and epithelial tissues are typically found in different steps of cicatrization (see Section 4.1, wound healing steps). However, the chronic wound microenvironment is heterogeneous, and the cicatrization steps may overlap each other, so it is possible to find both tissue types in the same wound bed [21].

Granulation tissue is formed in the proliferative phase of wound healing. It is characterized by the growth of new blood vessels and the deposition of extracellular matrix (ECM) components, such as collagen. Granulation tissue appears as red, beefy, and granular tissue that fills the wound bed. It provides support for the migration of cells involved in wound healing, such as fibroblasts, endothelial cells, and inflammatory cells [22,23].

The epithelium is responsible for covering and protecting the wound bed. Epithelialization begins in the later stages of wound healing, particularly during the remodelling phase. Epithelial cells at the wound edges start to migrate across the wound bed, closing the wound and restoring the epithelial barrier. These cells undergo proliferation, migration, and differentiation to form a continuous layer of epithelial tissue. The newly formed epithelium gradually thickens and matures, restoring the protective function of the skin and contributing to wound closure [22,23]. 

#### 3.1.3. Devitalized Tissue (Slough, Fibrinous Tissue)

Slough is the non-viable, yellowish, or white, stringy soft tissue that is often present in the chronic wound bed. It consists of dead cells, fibrin, debris, and bacteria. Slough is considered a barrier to healing as it can impair the formation of granulation tissue and hinder wound healing [24]. 

Based on the results of the International Wound Infection Institute’s case study series [24] on the slough, the most prevalent proteins detected in slough samples with low bacterial burden were associated with skin structure, wound healing, and immune responses. In chronic wounds with a high bacterial burden, proteins produced by bacteria were among the main proteins detected.

Effective wound management involves the removal of slough through debridement techniques to promote a clean and healthy wound bed, facilitating the progression towards healing. Methods for wound debridement include [12]:

- Surgical/sharp debridement;
- Mechanical debridement (washing solutions, whirlpool therapy, wet-to-dry dressings, ultrasound-assisted debridement, and lavage);
- Enzymatic debridement (topical application of enzymes breaks down the tissue, attaching necrotic tissue to the wound bed);
- Autolytic debridement (application of dressings facilitates the development of the body’s own enzymes to rid a wound of necrotic tissue);
- Biosurgical debridement (sterile larvae).

### 3.2. Wound Bed Temperature

Based on the review conducted by Gethin et al. (2021) [25], the chronic temperatures in the non-infected wound bed ranged from 30.2 °C to 33.0 °C, which is close to normal skin temperature. Specifically, the mean temperatures reported for different types of chronic wounds were 31.7 °C, 31.6 °C, 33.3 °C, 30.9 °C, and 32.0 °C for VLU, diabetic foot ulcers (DFUs), pressure ulcers (PUs), mixed arterial–venous ulcers, and ulcers of unknown aetiology, respectively. It is worth mentioning that in vitro studies have identified 33 °C as a critical temperature at which the activity of neutrophils, fibroblasts, and epithelial cells decreases.

In the study conducted by Dini et al. (2015) [26], a significant relationship between the wound bed score and the wound bed temperature was discovered. The findings from their study revealed a consistent increase in the wound bed score, indicating improved wound conditions, as the wound bed temperature increased. Furthermore, in acute wounds, it was observed that the inflammatory phase was associated with a temperature rise of approximately 2–3 °C compared to non-inflamed wounds. Subsequently, as wound healing progressed, the temperature tended to decrease.

Siah et al. (2019) [27] conducted a study on surgical wounds, both infected and non-infected, and found that there was a significant increase in temperature from days 1 to 4 post-surgery in both groups. Interestingly, the infected wounds exhibited a statistically significant lower temperature compared to the non-infected wounds, with higher periwound skin temperatures. These findings suggest that infection is associated with an inflammatory response.

### 3.3. Biochemical Composition of the VLU Microenvironment

**Figure 2:**
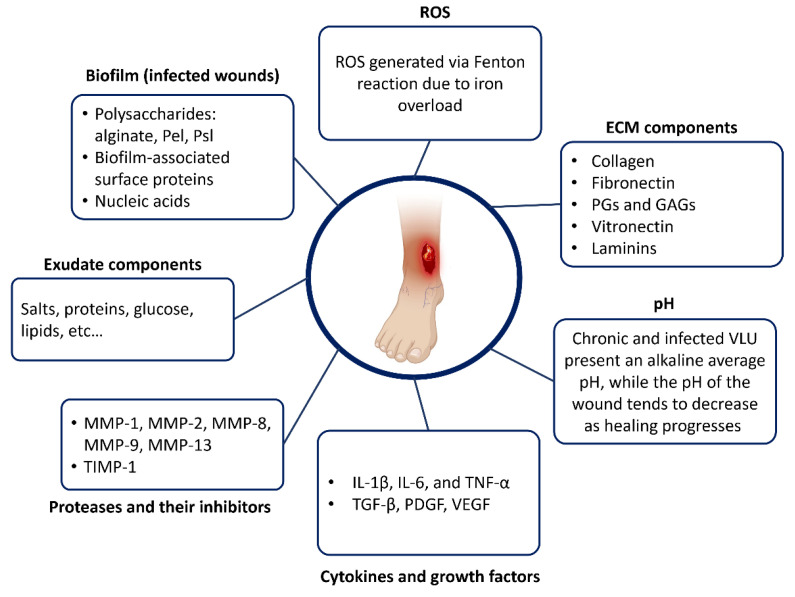
Major categories of biochemical compounds are found in the VLU microenvironment.

In this section, we will discuss the biochemical aspects of the VLU wound bed. By examining the different compounds present and their roles in the healing process, we aim to gain a better understanding of chronic VLU biochemistry. Figure 2 summarizes the complexity of chronic VLU biochemistry.

#### 3.3.1. Wound Bed pH

The normal pH of the skin ranges from 4.1 to 5.8, with skin in better conditions having pH values below 5.0. When an injury occurs, the skin’s pH level changes, leading to different impacts on the healing process. The exposed tissue pH is initially neutral due to disrupting the naturally acidic skin barrier and microcirculation. This less acidic environment persists in chronic wounds as a part of the chronicity process [28,29], resulting in reduced oxygen availability to the tissue, known as the Bohr effect. 

Changes in pH levels can also occur due to bacterial activity. Bacterial enzyme activity, such as urease produced by bacteria, like *P. mirabilis* and *P. aeruginosa*, can release ammonia, increasing pH levels. This environment can damage the tissue and further reduce tissue oxygenation, leading to potential complications in the healing process. Additionally, the alkaline wound environment can impair the regulation of metalloproteinases’ proteolytic activity, resulting in excessive degradation of extracellular matrix and growth factors, ultimately hindering the healing process [30].

The alkalinity of a wound has a significant impact on its healing potential, with higher alkalinity increasing the risk of non-healing by 8% [31]. Chronic VLU is characterized by an alkaline pH ranging from 7.9 to 8.7, along with bacteria such as *S. aureus* and *P. aeruginosa*. Interestingly, no direct association has been found between the bacterial profile and pH levels in these ulcers, although infection indeed raises the wound pH [30]. Additionally, for every 1-unit change in pH, a corresponding change in wound size of approximately 116.05 mm^2^ can be expected [32].

In a study conducted by Strohal et al. [33], a group of patients with highly colonized CLU, including VLU and other aetiologies, exhibited an initially high alkaline wound bed pH of 9.23 ± 0.61. As the researchers administered the antimicrobial treatment, both the wound bed pH and bacterial burden gradually decreased, consistent with findings from further studies [28,29]. Table 3 summarizes the pH changes in different stages of a chronic VLU.

#### 3.3.2. Extracellular Matrix Components

The extracellular matrix (ECM) is a complex network of molecules that surrounds and supports tissue cells, playing crucial roles in tissue structure and integrity, particularly for vascular walls and skin, so it is natural that they are present in the wound microenvironment [36,37]. The ECM is comprised of various components, which will be briefly discussed below. 

##### Collagens

Collagens are the most abundant proteins in the ECM and provide structural integrity, contributing to the mechanical properties, organization, and shape of tissues. They form fibrils and networks that give tissues their mechanical properties. Collagen interacts with cells through various receptor families, regulating their proliferation, migration, and differentiation processes [38]. 

Collagens consist of three polypeptide chains called alpha chains. The three alpha chains of fibril-forming collagens are organized in a triple helix structure with a one-residue shift between neighbouring chains [38]. The collagen family comprises 28 members that differ in their amino acid sequence, tissue distribution, and supramolecular organization. The most important types of collagen are as follows:

- Type I collagen: It is a fibrillar collagen composed of two alpha-1 chains and one alpha-2 chain. It forms a triple helix structure with a one-residue stagger between adjacent chains. Type I collagen is the most abundant type and provides tensile strength to tissues such as skin, bones, tendons, and ligaments.
- Type II collagen: It is also a fibrillar collagen but is composed of three identical alpha-1 chains. It forms a triple helix structure with a different stagger than type I collagen. Type II collagen is found in cartilage and provides structural support for this tissue.
- Type III collagen: It is a fibrillar collagen composed of three identical alpha-1 chains. It forms a triple helix structure with a different stagger than type I or II collagens. Type III collagen is found in blood vessels and internal organs and contributes to their mechanical properties.
- Type IV collagen: It is a non-fibrillar collagen that forms networks rather than fibers. It consists of three alpha-1 chains and three alpha-2 chains that form a triple helix structure with interruptions in the repeating sequence of amino acids. Type IV collagen provides structural support for basement membranes [38].

Collagen is constantly produced by fibroblasts and degraded by enzymes called matrix metalloproteinases (MMPs). This turnover process is essential for maintaining tissue integrity and repairing damaged tissues. In chronic wounds, collagen turnover can be impaired due to overexpression of MMPs (see Section 3.3.4; cytokines and growth factors) [38].

##### Fibronectin

Fibronectin is a glycoprotein that acts as a bridge between cells and the ECM. It plays a crucial role in cell adhesion, migration, and tissue remodelling. The matrix protein fibronectin is typically present at high concentrations in serum or plasma but undergoes degradation under inflammatory wound conditions, so it is present in lower concentrations in the chronic wound bed and/or exudate compared to serum and acute wounds [37,39,40].

##### Proteoglycans and Glycosaminoglycans

Proteoglycans (PGs) and glycosaminoglycans (GAGs) are important components of the extracellular matrix (ECM). PGs consist of a core protein to which one or more GAG chains are covalently attached. GAGs are long, highly negatively charged heteropolysaccharides that contain repeating disaccharides composed mainly of N-acetylated hexosamines and D-/L-hexuronic acid [41].

PGs and GAGs are present in the wound bed in various forms. During the early stages of wound healing, they contribute to the formation of a provisional matrix that provides a scaffold for cell migration and proliferation. In the wound bed, they interact with growth factors, cytokines, chemokines, cell surface receptors, and other ECM molecules to regulate cell behaviour during wound healing. PGs and GAGs are also present in various cell types involved in wound healing, such as fibroblasts and neutrophils [42].

##### Vitronectin

Vitronectin is an extracellular matrix protein that is involved in cell adhesion and migration. It is a glycoprotein that consists of two chains linked by disulphide bonds. Vitronectin has multiple binding sites for integrins, which are transmembrane receptors that mediate cell adhesion to the extracellular matrix. The interaction between vitronectin and integrins plays a crucial role in cell migration and tissue repair by modulating the activity of growth factors such as transforming growth factor-beta (TGF-β) and vascular endothelial growth factor (VEGF), which are involved in promoting ECM synthesis and remodelling, angiogenesis, and wound healing [41,42].

##### Laminins

Laminins are a family of glycoproteins that are present in the extracellular matrix (ECM) and play important roles in cell adhesion, migration, and survival. They consist of three polypeptide chains (α, β, and γ) that form a cross-shaped structure with globular domains at the ends of each arm [41].

#### 3.3.3. Proteases and Their Inhibitors

There is a sustained inflammatory reaction in VLU, where constant tissue damage from the underlying disease (CVD), microorganisms, and ECM fragment molecules constantly attract immune cells. Consequently, the pro-inflammatory cytokine cascade is amplified and persists for extended periods, leading to higher protease levels [43].

Matrix metalloproteinases (MMPs) are a group of enzymes found in the extracellular space that contribute to the breakdown of ECM components. They exert influence over cell migration, adhesion, and interactions between cells. Additionally, MMPs have been extensively studied in the context of wound healing and ulcer treatment, demonstrating their potential significance across various wound types and cutaneous ulcers [44].

MMPs are regulated by their natural inhibitors, the tissue inhibitors of metalloproteinases (TIMPs) [36]. Excessive MMP activity has been associated with impaired wound healing and chronicity. In 1996, Trengove et al. [45] reported the differences in MMP wound fluid concentrations in healing versus non-healing wounds, highlighting the potential of MMPs as biomarkers for wound healing outcomes. Since then, several studies have investigated using MMP analysis to predict the healing outcomes of chronic wounds, including venous leg ulcers (Table 4). 

Healing VLUs tend to have lower levels of most MMPs, regardless of the type of treatment applied. Conversely, non-healing VLUs, including those infected or not yet treated, tend to exhibit higher levels of most MMPs. These results suggest that reducing MMP activity is crucial for VLU healing, while high MMP activity is associated with poor healing outcomes [45].

Among the MMPs studied in most articles about chronic VLU, MMP-1, MMP-2, and MMP-9 were the most investigated, with the most significant results. Together, these MMPs play a critical role in the breakdown of interstitial collagen types I, II, III, and IV and other extracellular matrix components [38]. Collagen turnover in chronic wounds is believed to be impaired by overexpression of MMPs, particularly MMP-8, MMP-13, and MMP-9, which affect tissue remodelling, re-epithelialization, and collagen fiber integrity [44].

#### 3.3.4. Cytokines and Growth Factors

Cytokines are small proteins released by cells that have a specific effect on the interactions and communications between cells [51], and growth factors (GFs) are soluble proteins that regulate and direct the process of wound healing [52]. Several cytokines and growth factors have been identified as critical players in wound healing and are commonly found in biochemical analyses performed on different components of the wound bed, especially exudate and tissues. Some of the important cytokines and growth factors include:

- Transforming Growth Factor-beta (TGF-β): TGF-β is a growth factor involved in inflammation, cell migration, angiogenesis, proliferation, collagen production, and differentiation. It is a key regulator of epithelial–mesenchymal transition (EMT), a process by which epithelial cells lose their cell–cell adhesion and apical–basal polarity and acquire a mesenchymal phenotype [52]. It is a key regulator of epithelial–mesenchymal transition (EMT), a process by which epithelial cells lose their cell–cell adhesion and apical–basal polarity and acquire a mesenchymal phenotype. Among the three isoforms of TGF-b (TGF-β1, TGF-β2, and TGF-β3), TGF-β2 has been shown to play a specific role in EMT in various tissues [53].
- Platelet-Derived Growth Factor (PDGF): this GF triggers the formation of granulation tissue at the wound site and tissue regeneration processes including chemotaxis, fibroblast proliferation, and collagenase production [52].
- Vascular Endothelial Growth Factor (VEGF): VEGF is a potent inducer of angiogenesis, promoting the formation of new blood vessels in the wound bed [54].
- Interleukin-1β (IL-1 β): IL-1β is a pro-inflammatory cytokine that regulates inflammation and immune responses. IL-1β is primarily released by certain immune cells like monocytes and macrophages, as well as nonimmune cells including fibroblasts and endothelial cells, in response to cellular injury, infection, invasion, and inflammation [51].
- Interleukin-4 (IL-4): IL-4 programs macrophages to down-regulate pro-inflammatory mediators and to promote wound healing processes by contributing to the production of extracellular matrix and by activating fibroblasts [55].
- Interleukin-6 (IL-6): IL-6, a pro-inflammatory cytokine, is an important mediator of protein catabolism in infectious diseases such as infected VLUs [36]. IL-6 is involved in the acute phase of the response to injury or infection. It has been shown to convert naïve T-cells into CD4þ/CD25þ T Reg or IL-10-secreting CD4þ T-cells (Tr1) [55].
- Interleukin-10 (IL-10): IL-10 is an immunoregulatory cytokine that plays a role in modulating the immune response to support wound healing. IL-10 is discussed in this article as an important immunoregulatory cytokine that exhibits both suppressive and stimulatory effects on the immune system. It acts in an anti-inflammatory manner on macrophages and DCs by reducing the production of pro-inflammatory cytokines and presenting antigens to T-cells [55].
- Tumour Necrosis Factor-alpha (TNF-α): TNF-α is a pro-inflammatory cytokine involved in different cellular processes, including inflammation, cell proliferation, and apoptosis. It influences the early stages of wound healing and can contribute to chronic inflammation when dysregulated, which is associated with pain [51].

Activated macrophages, as the primary producers of proinflammatory cytokines that contribute to the activation of inflammatory reactions [51], play a crucial role in the development of pain in chronic VLU patients. Studies have shown the involvement of specific pro-inflammatory cytokines, such as IL-1β, IL-6, and TNF-α, in the pathogenesis of pain in these patients [51,56]. 

VLUs are characterized by elevated levels of IL-1 alpha, IL-1β, and TNFα, along with increased protease activity, which contributes to the sustained inflammatory state observed in VLUs. These biomarker levels (among others) in chronic VLU exudate were evaluated in a study conducted by McQuilling et al. in 2021 [46], where the amniotic membrane was applied as a treatment. Table 5 shows the levels of cytokines found in this study. 

#### 3.3.5. Reactive Oxygen Species (ROS) and Antioxidants

In late-stage CVD, erythrocytes escape into the extracellular space surrounding skin tissue due to venous hypertension and valve and vein wall damage. In response, macrophages phagocytose the erythrocytes, releasing iron that binds to intracellular ferritin and eventually forms hemosiderin. As a result, the iron concentration in the lower limbs of VLU patients can be up to 20 times higher than that in their upper arms. In the presence of hydrogen peroxide, released by activated macrophages and neutrophils, iron can promote the generation of highly toxic hydroxyl radicals via the Fenton reaction [15,58]. This cascade of events triggers a heightened activation of proteases and inflammatory cytokines [43].

Oxidative stress can result in DNA damage, leading to cell cycle arrest or abnormal metabolic changes. These changes are directly associated with the senescence observed in various cell populations, including keratinocytes and fibroblasts, in VLU. Senescent cells are unresponsive to typical wound healing signals, with senescent fibroblasts exhibiting reduced proliferative capacity that correlates with wound healing failure [43].

ROS scavenging is a strategy employed in some newly developed wound dressings. For example, a cross-linked polyphenol/polysaccharide hydrogel, developed by Qi et al. [59], enhanced wound healing by scavenging intracellular ROS at the cellular level.

#### 3.3.6. Wound Exudate Composition as a Function of the Physiological Condition

Wound exudate, derived from blood serum, serves as the primary source of nutrients for cells within the wound bed, sharing similarities in composition with salts, glucose, and other proteins [5,10,18,20,37,60]. However, it also contains specific components absent in serum, such as ECM proteins (vitronectin and fibronectin), and it may contain molecules produced by bacterial biofilms in the case of infected wounds (see Section 4.2). A comparison of the composition of healing and non-healing wound fluid to serum can be found in Table 6 [20,45,61,62,63]. 

While recent studies have focused on biomarkers in wound fluid, there remain a limited number of studies that have examined wound fluid at a biochemical level [20,45,61,62,63]. The biochemical composition of wound fluid can vary among patients, influenced by factors such as the patient’s general health, nutritional status, wound type, size, and phase of healing. Table 7 provides a comparison of healing and non-healing exudates from VLU. Additionally, the previous management of the wound bed may also impact the composition of wound fluid samples [18,20]. 

## 4. Chronic VLU Progression: Healing and Infected Stages

Despite advancements in wound care, not all VLUs heal at the same rate, and some may not heal, becoming chronic VLUs. In this chapter, we will discuss two different scenarios in VLU progression: wound healing and wound infection, which is one of the main causes of delayed healing in all types of chronic wounds.

### 4.1. Overview of the Wound Healing Steps

#### 4.1.1. Haemostasis

Hemostasis occurs immediately following an injury, and it is considered a part of the inflammatory phase by some authors. The injured area quickly attracts platelets. When the damaged blood vessel exposes collagen, it triggers their activation and subsequent release of ADP and glycoprotein adhesion molecules such as fibrinogen, fibronectin, vitronectin, thrombospondin, and von Willebrand’s factor, which cause the platelets to aggregate [64,65].

When platelets activate prothrombin, it transforms into thrombin, catalyzing the conversion of fibrinogen into fibrin, thus creating a thrombus that serves as a provisional matrix. Fibronectin, a protein containing various integrin receptors, is vital in stimulating the migration and adhesion of fibroblasts, keratinocytes, and endothelial cells [64].

In addition to their role in aggregating thrombus, platelets also possess integrin receptors, which contribute to further aggregation. Platelets also release growth factors such as PDGF, TGF-a, TGF-b, basic fibroblast growth factor (bFGF), insulin-like growth factor-1 (IGF-1), and vascular endothelial growth factor (VEGF), all of which are essential compounds in the recruitment of inflammatory cells [64].

#### 4.1.2. Inflammation 

During this stage, neutrophils and monocytes/macrophages are recruited to the injury site and release chemokines, cytokines, and growth factors. Neutrophils are attracted to the wound through the impact of chemokines, such as thrombin, leukotrienes, PDGF, TGF-b, and bacteria, as well as the complement system, to organize a defense against infection [64].

After 24 h, neutrophils undergo apoptosis, and macrophages take over, leading to further inflammation and phagocytosis. Macrophages secrete cytokines and growth factors, such as IL-1, IL-6, IL-8, TNF-a, TGF-b, PDGF, TGF-a, bFGF, HB-EGF, and various MMPs (1, 2, 3, 9, and 10), to help clear cellular debris and recruit fibroblasts for collagen and matrix deposition. Additionally, VEGF, EGF, and PDGF direct the initiation of angiogenesis [64,65].

#### 4.1.3. Proliferation

The objectives during the proliferative phases are the replacement of the provisional fibrin matrix with a new matrix of collagen fibers, proteoglycans, and fibronectin to restore the structure and function of the tissue. Additionally, angiogenesis, the development of new capillaries to replace damaged vessels and restore circulation, is also a crucial event in the healing process. Another significant event is the formation of granulation tissue and epithelialization. During this phase, fibroblasts play a critical role as the main cells involved in the healing process [65].

During the proliferative phase, fibroblasts play a critical role in wound healing by depositing collagen, elastin, proteoglycans, glycosaminoglycans, and non-collagenous glycoproteins to replace the provisional matrix. Various growth factors, such as PDGF, EGF, IGF-1, and TGF-b, regulate these fibroblasts. Collagen deposition (types I and III) increases up to the third week and then begins to decline [64].

During the proliferation phase, angiogenesis also occurs, which involves forming new blood vessels in the injured area. This process is regulated by angiogenic factors such as VEGF, PDGF, and MMPs. Endothelial cells form new capillaries for oxygen, carbon dioxide, and nutrient transport [64].

At the same time, epithelialization occurs, which involves the detachment of keratinocytes from the wound edge and skin appendages (sweat glands and hair follicles). These keratinocytes then migrate, proliferate, and cover the collagen-rich matrix. This process is regulated by growth factors, such as TGF-a, EGF, KGF, and TGF-b, as well as matrix metalloproteinases, such as MMP-2, MMP-9, MMP-1, MMP-3, and MMP-10 [64,65].

The proliferative phase is a critical step in wound healing, where fibroblasts, epithelial cells, and endothelial cells work together to deposit extracellular matrix proteins, promote epithelialization, and restore blood vessels in the injured area [64].

#### 4.1.4. Remodelling

The final phase of the healing process is called remodelling. During this phase, the granulation tissue matures into scar tissue, and the tensile strength of the tissue increases. As the granulation tissue matures, the number of capillaries decreases, and the amount of glycosaminoglycans and water also decreases. The cell density and metabolic activity in the granulation tissue also decrease during this phase [65]. 

Changes in the type, amount, and organization of collagen occur during remodelling, enhancing the tissue’s tensile strength. Initially, type III collagen is synthesized at high levels, but it is eventually replaced by type I collagen, the dominant fibrillar collagen in the skin. Notably, the tensile strength of a newly epithelialized wound is only about 25% of that of normal tissue, and healed or repaired tissue is never as strong as normal tissue that has never been wounded.

Tissue tensile strength is mainly enhanced by the reorganization of collagen fibers deposited randomly during granulation and increased covalent cross-linking of collagen molecules by the enzyme lysyl oxidase, which is secreted into the extracellular matrix by fibroblasts. Over several months or more, changes in collagen organization in the repaired tissue slowly increase the tensile strength to a maximum of about 80% of normal tissue.

During the remodelling phase, the extracellular matrix proteins are also remodelled through the actions of several different classes of proteolytic enzymes produced by cells in the wound bed at different times during the healing process. MMPs and serine proteases are two of the most important families of these enzymes. 

Specific MMP proteases that are necessary for wound healing are the collagenases, which degrade intact fibrillar collagen molecules; the gelatinases, which degrade damaged fibrillar collagen molecules; and the stromelysins, which effectively degrade proteoglycans. Another important serine protease is neutrophil elastase, which can degrade almost all types of protein molecules [64,65].

### 4.2. Infection and Its Impact on the Wound Microenvironment

Delayed healing is often caused by infection, which can prolong the inflammatory phase and lead to further tissue damage. At least 60% of chronic wounds, including VLU, are infected. Bacterial colonization is a significant factor in VLU chronicity. Even though it may not initially cause a harmful immune response, the microorganisms can express virulence factors, such as the development of biofilms. These biofilms can be tolerant of the host’s defense mechanisms, thus creating a risk for infections [30].

According to the studies conducted by Serra et al. [36], Rabello Sergio et al. (2022) [30], and Thomsen et al. (2010) [66], the most common bacteria found in infected venous ulcers include *Staphylococcus aureus*, *Pseudomonas aeruginosa*, and *Corynebacterium striatum*. *S. aureus* and *P. aeruginosa* were identified as the most frequently observed bacterial species in venous ulcers, according to both Serra et al. (2014) and Rabello Sergio et al. (2022) [30] studies. Thomsen et al. (2010) [66] also reported that *S. aureus*, *P. aeruginosa*, and *Enterococcus faecalis* were frequently observed in chronic VLU when culture-based methods were used. However, the diversity of bacteria in venous ulcers is generally polymicrobial and heterogeneous, as Thomsen et al. (2010) [66] noted. *Proteus mirabilis* was also found to be a commonly identified bacterial species in venous ulcers, according to the study by Rabello Sergio et al. (2022) [30].

Antibacterial wound dressings are crucial for managing infected wounds. Silver is widely used for its proven antimicrobial properties, including against antibiotic-resistant bacteria [67]. Newer strategies have also emerged, such as octenidine dihydrochloride as an antimicrobial agent [68] and dressing materials with antibacterial activity. For instance, Cheng et al. [69] have developed hydrogels that can electrostatically kill *Staphylococcus aureus* and *Escherichia coli*, promising improved wound healing.

It is important to note that several commensal bacteria have been found to be beneficial for wound healing, including *Staphylococcus epidermidis*, *Lactobacillus plantarum*, *Lactobacillus fermentum*, *Saccharomyces cerevisiae*, *Lactobacillus reuteri*, and *Bacillus subtilis* sp. *natto*. These bacteria can help prevent pathogen invasion, produce antimicrobial peptides, limit inflammation, induce commensal-specific T-cell responses, and promote tissue repair. However, the effects of probiotics and postbiotics on wound healing are still being studied, and more research is needed to fully understand their potential benefits [70].

#### 4.2.1. The Pathogenesis of Wound Infection

**Figure 3:**
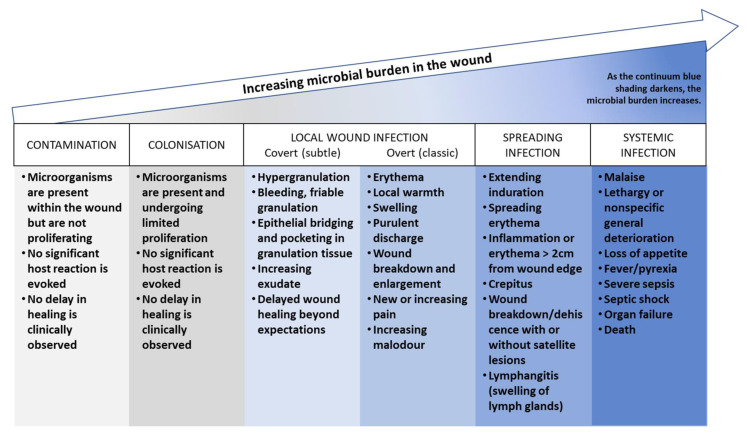
Wound Infection Continuum according to the International Wound Infection Institute [71].

Bacteria are present in the wound bed from the initial stages of wound formation. The microbial burden in the wound grows over time, as shown in Figure 3, following a continuum. The International Wound Infection Institute (2022) [71] has defined different stages of the wound infection continuum as follows:

- *Contamination:* this stage refers to the presence of non-replicating microorganisms on the surface of a wound. These microorganisms may be introduced during dressing changes or other procedures, but they do not necessarily cause an infection.
- *Colonization:* this stage refers to the presence of replicating microorganisms on the surface of a wound. These microorganisms are able to multiply and form biofilms, but they do not necessarily cause an infection.
- *Local infection (covert and overt stages):* this stage refers to the presence of replicating microorganisms that have caused damage to host tissues and are causing an inflammatory response. The covert stage is characterized by subtle clinical indicators that suggest an infection is present, while the overt stage is characterized by classic signs and symptoms of local wound infection, such as erythema, warmth, pain, and purulent discharge.
- *Spreading infection:* this stage refers to the spread of microorganisms beyond the local area of tissue damage into adjacent tissues or structures.
- *Systemic infection:* this stage refers to the spread of microorganisms beyond the local area of tissue damage into the bloodstream or other body systems, leading to sepsis or other systemic complications.

#### 4.2.2. Biofilm Formation

**Figure 4:**
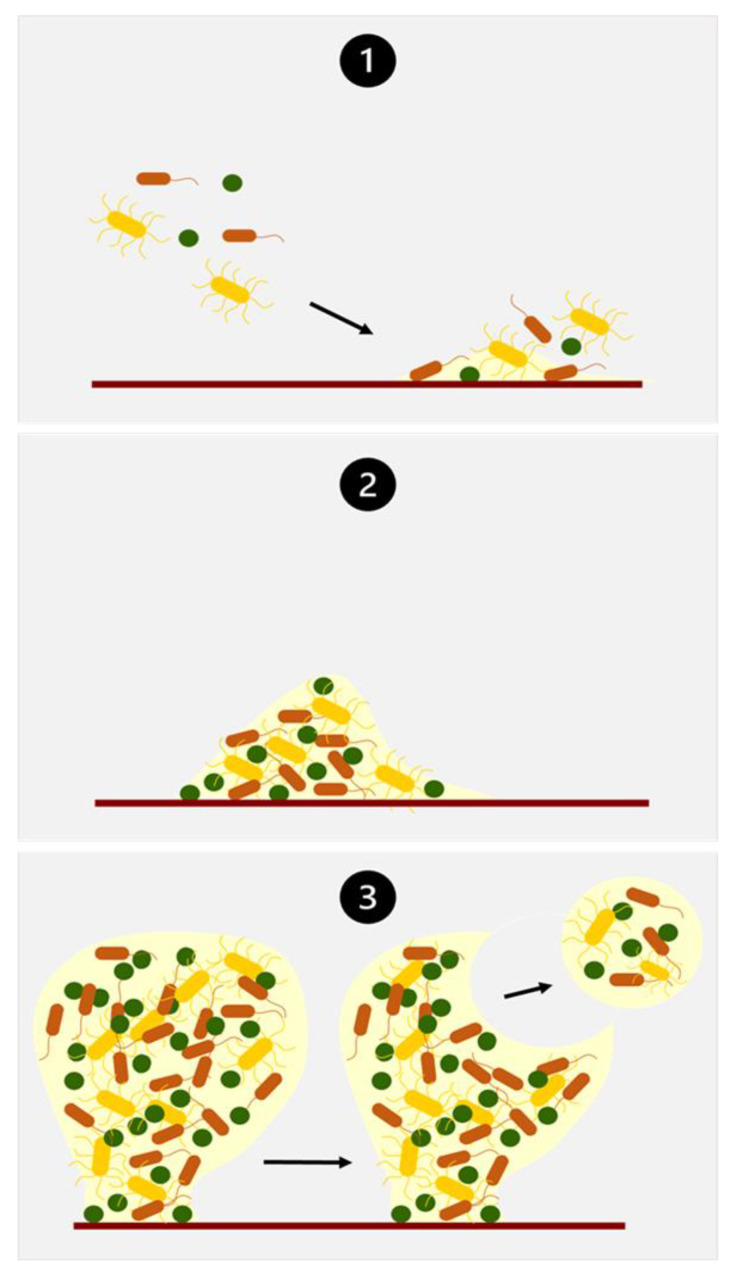
Mechanism of biofilm formation in wounds [60,73].

The wound microenvironment is moist and rich in proteins, salts, and glucose, so it is an ideal environment for the attachment and growth of microorganisms. Bacteria may exist in a wound bed (as on different surfaces) in two states: a free-living or planktonic state and an attached or sessile state called biofilm, in which microorganisms are attached to each other. The stages of biofilm development are similar in various circumstances and microbial types [60,72]. In the context of wound biofilms, the process can be categorized into three primary stages (Figure 4).

The initial stage involves the attachment of microorganisms to the wound surface (Stage 1). Once attached, microorganisms begin the production of an extracellular polymeric substance (EPS) that enhances and strengthens their attachment. This EPS also promotes adhesion and serves as a nutrient source for growth. The initial colonizing microorganisms modify the microenvironment, facilitating the recruitment of subsequent bacteria (Stage 1). As microbial cells multiply, they form clusters or microcolonies (Stage 2). During this phase, EPS production intensifies, aiding in the aggregation of microbes within the biofilm structure [60,73].

Over the course of several weeks or months, the developing biofilm reaches a quasi-steady state. Notably, three-dimensional mushroom-shaped structures begin to emerge (Stage 3) within in vitro biofilms, reflecting increased complexity in their organization. Microbial cells within the biofilm employ detachment and dissemination mechanisms as they strive to colonize new surfaces. These processes encompass shedding, detachment, or shearing mechanisms [60,73].

#### 4.2.3. Biofilm Biochemistry

The main components of biofilm are extracellular polymeric substances (EPS), which are mainly composed of polysaccharides, proteins, nucleic acids, and lipids. These biopolymers provide the mechanical stability of biofilms, mediate their adhesion to surfaces, and form a cohesive, three-dimensional polymer network that interconnects and transiently immobilizes biofilm cells [72].

EPS, which can make up 50% to 90% of the total organic carbon in biofilms, serves as the primary matrix material. EPS can vary in its chemical and physical properties, but it is predominantly composed of polysaccharides. These polysaccharides can be neutral or polyanionic (in the case of Gram-negative bacteria), with the anionic nature conferred by the presence of uronic acids (e.g., D-glucuronic, D-galacturonic, and mannuronic acids) or ketal-linked pyruvates. This anionic property is important because it allows the association of divalent cations like calcium and magnesium. These cations can cross-link with the polymer strands, leading to increased binding strength within a mature biofilm [60,74].

##### *Pseudomonas aeruginosa* Biofilm

*Pseudomonas aeruginosa* biofilm is one of the best-studied models for biofilm formation. Alginate, Psl, and Pel are three of the main polysaccharides produced by *P. aeruginosa* [72,75]. Little is known about alginate concentrations in the wound exudate; however, local concentrations of alginate in a *P. aeruginosa* biofilm may approach 5 mg/mL and 0.004–0.1 mg/mL in cystic fibrosis sputum [76]. *P. aeruginosa* is also capable of secreting proteases, such as elastase, to inactivate parts of the complement system [73].

##### *Staphylococcus aureus* Biofilm

*Staphylococcus aureus* biofilm is less well characterized than *P. aeruginosa*. A group of extracellular proteins called biofilm-associated surface proteins (BAPs) are commonly found in *S. aureus* biofilm. These are high-molecular-mass proteins on the bacterial cell surface that promote biofilm formation. They contain a core domain of tandem repeats that are required for the formation of a biofilm and play a part in bacterial infectious processes [72].

#### 4.2.4. Impact of Infection on Exudate Characteristics

The nature and volume of exudate in a wound can undergo changes when an infection occurs (Table 8). Generally, an increase in exudate volume is associated with infection. For instance, if the infection is primarily caused by *Pseudomonas aeruginosa*, the exudate can become thicker and have a greenish or blue color [20]. However, the characteristics of exudate may vary depending on the species involved. For example, bacteria like *Staphylococcus aureus*, which produce fibrinolysis, are associated with thin and serous exudate produced in higher amounts [21].

It is worth emphasizing that components of the biofilm can be found in the wound exudate. When microbial cells within the biofilm seek to colonize new surfaces, they undergo detachment and dissemination processes, which can occur through shedding, detachment, or shearing mechanisms. Because of these changes, clumps containing thousands of bacterial cells are formed within the biofilm and subsequently reintroduced into the wound exudate present in the wound bed. Note that these released cells retain the distinct traits and characteristics of the original biofilm, effectively preserving their biofilm properties before establishing themselves on a new surface [60].

## 5. Impact of the Wound Microenvironment on Wound Care Products

### 5.1. Simulated Conditions

Conducting performance and safety tests is essential for assessing medical devices and obtaining CE markings. Likewise, in the research field, different tests are needed during the development of new biomaterials or medical devices. Whether the tests are performed by a company or researchers, the biological conditions in which the product will be employed must be simulated.

Wound dressings for chronic wound care, including VLU, are medical devices in contact with injured skin. Depending on their composition, they are classified as IIa, IIb, or III according to the new European Medical Devices Regulation (MDR), fully applicable since 2021, which replaces the previous Medical Devices Directive (MDD) [80]. Beyond performance tests such as absorbency, moisture vapour transmission rate (MVTR), and fluid handling capacities, classes II and III now need to be tested in terms of extractables and leachables as part of biological risk assessment.

All the above-mentioned tests require the use of simulated exudate or simulated wound fluid (SWF) to be performed. However, there is no standard for a SWF, and different compositions can be found in the literature. Here, we will briefly discuss some of the SWFs employed for wound dressing assessment. Table 9 summarizes the composition of these SWFs.

#### 5.1.1. Saline SWF

These SWFs are proposed by standards, such as NF EN 13726-1/2002 [81], and they are mainly used in absorption or swelling tests. They claim to have a similar ionic strength to the wound exudate. However, this type of SWF fails to represent the complexity of wound fluid concerning pH, biochemical composition, and viscosity. Forss et al. (2022) [82] investigated the impact of the SWF viscosity on the absorption rate. Their results indicated that the viscosity of the solution alone has a significant effect on the absorption time and that the combined effect of dressing type and viscosity influences absorption.

#### 5.1.2. SWF Containing 2% Bovine Serum Albumin

This type of SWF is one of the most commonly used in wound dressing assessment [83,84,85,86,87], especially in research. It is an easily prepared, accessible, and reproducible SWF. Although more representative of wound fluid, it still lacks many crucial features found in actual wound fluid, including vital proteins and lipids.

#### 5.1.3. FBS-Based SWF

Foetal bovine serum (FBS) shares many similarities in composition with wound exudate. It already contains serum components like albumins, proteins, immunoglobulins, lipids, minerals, vitamins, and potentially growth factors [5,37]. One advantage of FBS is its easy availability. However, as a biological product, it exhibits batch-to-batch variability that researchers cannot control, which may affect the results depending on the context of its use. Additionally, FBS lacks certain extracellular matrix (ECM) components. To address this limitation, Kadam et al. [37] supplemented FBS with commonly found wound exudate components, such as collagen and vitronectin, to produce a SWF.

#### 5.1.4. Other SWF Approaches

Lutz et al. [78] employed a different approach to developing a SWF. To prepare the SWF, several components were added to PBS. These included whey protein isolate to simulate protein content, vegetable oil to incorporate lipids, sodium bicarbonate for buffering purposes, simethicone as an anti-foaming agent, and dextrose/sucrose to provide sugar content. The authors obtained a reasonably accurate composition of SWF compared to the literature [45]. However, the addition of components that are not present in wound exudate may be an issue depending on the application of this SWF.

Hobson et al. [88] used a SWF containing albumin, globulins, fatty acids, and triglycerides for testing their new absorbent material. This is an interesting composition that better represents the main classes of biochemical compounds found in wound exudate. However, no details about the triglycerides and fatty acids used were found. Also, we should expect solubility problems when employing hydrophobic components in an aqueous solution.

**Table 9:** SWF compositions.

SWF Composition	Applications in Wound Dressing Assessment	References
2% BSA 0.02 M 0.02 M calcium chloride 0.4 M sodium chloride 0.08 M tris-aminomethane Dissolved in deionized water Adjusted pH 7.5	Swelling studies, water absorption and equilibrium water content, evaporative water loss, and adhesion studies of a newly formulated wound dressing. Release of active molecules/drug dissolution.	[83,84,85,86,87]
50% foetal calf serum and 50% maximum recovery diluent (0.1% *w*/*v* peptone [beef protein extract] and 0.9% *w*/*v* sodium chloride)	Investigation of the rate of silver release and antibacterial activity.	[67]
Foetal bovine serum mixed with an equal amount of peptone water	Development of a test method for measuring silver release from silver-containing dressings that are not fully saturated with test fluid.	[89]
Filtered foetal bovine serum	Interaction of biological fluids with materials used for wound healing applications. Changes in osmolality and total protein content were evaluated after contact with wound dressing material.	[5]
British Pharmacopoeia Solution A (142 mM NaCl, 2.5 mM CaCl.2H_2_O in distilled water)	Development of a new measurement system for real-time monitoring of moisture levels.	[90]
8298 g of NaCl (142 mM) 0.368 g of CaCl_2_ (3.32 mM) in deionized water making up to 1 L	Evaluation of certain aspects relating to the absorbency of dressings in contact with the wound.	[81,91]
Foetal calf serum (70%) Lactic acid (11–12 mM) Lactoferrin (20–30 μg/mL) Fibrinogen (200–400 μg/mL) Fibronectin (30–60 μg/mL) Collagen (10–12 μg/mL)	The study of planktonic growth, biofilm features, and interspecies interactions of *Staphylococcus aureus* and *Pseudomonas aeruginosa.*	[37]
Sodium (3.24 g/L) Potassium (0.56 g/L) Calcium (0.05 g/L) Magnesium (0.01 g/L) Chloride (3.40 g/L) Whey protein isolate (40.06 g/L) Vegetable oil (2.32 g/L) Sugars (1.05 g/L) Simethicone (0.04 g/L) Dissolved in PBS	Development of a new in vivo test method to compare wound dressing fluid handling characteristics and wear time.	[78]
0.2% *w*/*v* fatty acids, 4.0% *w*/*v* albumin, 2.5% *w*/*v* globulins 0.05% *w*/*v* triglycerides Dissolved in PBS	Absorption studies of a novel water-free hydrophilic absorbent for wound dressing application.	[88]

### 5.2. Examples of Impact and Interactions

The composition of the exudate (or SWF) in contact with wound dressings substantially influences the degradation and fate of the polymeric matrix and the compounds dissolved within the dressing [8]. Even minor alterations in the exudate composition can have significant consequences. Consequently, it is crucial to consider the impact of changes in the wound microenvironment when designing new dressings. 

For example, silver-containing wound care products rely on the generation of silver ions as the active antimicrobial component. One of the primary ions found in wounds is chloride, which exists in significant concentrations in wound exudate. Since silver chloride has low solubility in water, the excess of chloride ions alone effectively limits the release of silver ions to the aqueous solubility limit of silver chloride [8]. 

Proteins also play a crucial role in regulating the presence of free silver ions at the interface between the wound surface and the dressing. Silver ions have a strong affinity for proteins, peptides, amino acids, and cell surface structures of both host and microbial cells. The presence of 5% bovine serum albumin substantially increases the availability of silver from silver dressings [8].

A deep understanding of the wound microenvironment is also needed for the development of innovative wound dressings designed to accelerate healing [92]. Notable innovations include the development of responsive dressings and biomolecule-loaded dressings [7,85,93]. 

#### 5.2.1. Responsive Dressings

Responsive dressings, also known as smart or intelligent wound dressings, are designed to respond to specific conditions or changes in the wound environment. For example, Fan et al. (2019) [7] developed a pH-responsive hydrogel with a tobramycin combined effect for wound dressing applications. Tobramycin crosslinks dialdehyde carboxymethylcellulose through imine bonds, forming a hydrogel. In the weakly acidic environment of the wound, imine bonds break, leading to the release of the active molecule and moisture encapsulated within the hydrogel. At higher pH values, hydrolysis does not take place.

Another example of responsive dressing was developed by Rui et al. [94]. ROS-responsive hydrogels were prepared by modifying two natural polymers, namely sodium alginate and sodium hyaluronate, with 3-aminophenylboric acid. These modified polymers were subsequently cross-linked with polyvinyl alcohol to create the desired hydrogel formulation. The developed dressings were loaded with doxycycline, which was released in a controlled manner for 10 h due to the sensitivity of the boronate ester bonds to ROS.

#### 5.2.2. Biomolecule-Loaded Dressings

An area of current investigation in wound dressing research is the use of biomacromolecules such as growth factors [95]. However, its efficacy depends on exudate composition since each protein exhibits optimal stability and functionality within specific pH ranges. Deviation from this range can induce denaturation, resulting in the loss of native structure and function. Changes in pH disrupt the electrostatic interactions between charged amino acids, affecting the overall structure of the proteins. Denatured proteins may exhibit altered biological activity, highlighting the importance of the wound microenvironment [93].

## 6. Conclusions

Knowledge of the potential changes in the biochemical composition of exudates as a function of their stages is useful for effective wound care and assessing the performance of wound dressings. 

This review has emphasized the diverse compounds involved in changes, including extracellular matrix molecules, cytokines, growth factors, proteases, and reactive oxygen species. It has been highlighted that during different stages, such as chronic, healing, and infected phases, the biochemical composition of the chronic wound microenvironment undergoes significant changes. This knowledge holds importance not only for wound care but also for advancing wound dressing research.

However, it is worth noting that very few studies have explored the variations in simulated wound fluid. Further research is needed to comprehensively characterize the biochemical aspects of chronic wound biochemistry across various pathophysiological contexts, including infected wounds. The current SWF formulations do not fully represent the complexity of wound exudate nor account for the influence of observed composition changes within the context of dressing–wound interactions.

To the authors of this review, the lack of information and understanding regarding the evolution of exudates and their impact on wound care therapies could be solved in part by:

- Developing more sensitive and specific analytical methods by resorting, for instance, to microfluidic-based approaches to improve the characterization of the content of wound exudate while reducing the amount of wound exudate required;
- Applying these methods to exudates obtained from large patient cohorts with the view of gaining a better understanding of the variability of wound exudate and its changes in composition as a function of the stage of the wound;
- Proposing enhanced approaches to better highlight the mutual interactions between the exudate and the wound care therapies.

## Figures and Tables

**Table 1:** Clinical class, Etiology, Anatomy, Pathophysiology classification system [13].

**Clinical classification**
C0—No visible or palpable signs of venous disease
C1—Telangiectasias or reticular veins
C2—Varicose veins
C2r—Recurrent varicose veins
C3—Edema
C4—Changes in skin and subcutaneous tissue secondary to CVD
C4a—Pigmentation or eczema
C4b—Lipodermatosclerosis or atrophie blanche
C4c—Corona phlebectatica
C5—Healed
C6—Active venous ulcers
C6r—Recurrent active venous ulcer
**Etiologic classification**
Ep—Primary
Es—Secondary
Esi—Secondary-intravenous
Ese—Secondary-extravenous
Ec—Congenital
En—No cause identified
**Anatomic classification**
As—Superficial
Ad—Deep
Ap—Perforator
An—No venous anatomic location identified
Pathophysiologic classification *
Pr—Reflux
Po—Obstruction
Pr,o—Reflux and obstruction
Pn—No pathophysiology identifiable

* The presentation of the pathophysiological category should be supported by the appropriate anatomical location. The detailed anatomical classification can be found in reference [13].

**Table 2:** Main characteristics as a function of exudate type [20].

**Serous**	Clear or slightly yellow fluid is typically seen in the early stages of wound healing.
**Sanguineous**	Red and contains red blood cells, indicating damage to blood vessels.
**Serosanguineous**	Consisted of a mixture of serous and sanguineous exudate.
**Purulent**	Thick and opaque with a yellow or green colour, indicating the presence of infection.

**Table 3:** pH changes during different wound stages.

Unwounded Skin	Healing Wound	Chronic Wounds	Highly Infected Wounds
4.5–6.0 [34]	6.5–8.5 [35]	VLU: 7.9–8.7 [30] Chronic wounds in general: 7.2–8.9 [35]	8.64–9.86 [33]

**Table 4:** Different levels of MMPs and TIMPs in non-healing VLU versus healing VLU or acute wounds. The results from McQuilling et al. (2021) [46] are expressed in mean (confidence interval); the results from Ligi et al. [47] and Subramanian et al. [48] are expressed in mean ± standard deviation. The other results are expressed as mean values.

MMP/TIMP	Non-Healing VLU (pg/mL)	Healing VLU (or Acute Wounds for Some Studies) (pg/mL)	References
MMP-1	79,460 ± 26,370	142,800 ± 26,370	[47]
	100,000 ± 10,000	60,000 (acute graft)	[48]
	500,000	~150,000	[49]
MMP-2	943,900 ± 119,600	414,700 ± 65,300	[47]
	251,000 ± 101,000	178,000 ± 84,000	[50]
MMP-3	11,000 ± 3	8000 ± 4000 (acute graft)	[48]
	623.6 ± 165.8	3072 ± 1076	[47]
MMP-8	6,000,000	400,000	[49]
MMP-9	483,100 ± 68,190	173,900 ± 47,060	[47]
	293,000 ± 96,000	219,000 ± 113,000	[50]
MMP-10	4158 (2274, 6042)	6910 (5234, 8585)	[46]
MMP-12	67,550 ± 12,350	22,780 ± 7478	[47]
MMP-13	3093 ± 930.3	10,290 ± 3775	[47]
TIMP-1	2500	29,000 ± 2000 (acute surgical wound)	[48]
TIMP-4	2.7 (0.6, 6.1)	41.2 (19.8, 63.0)	[46]

**Table 5:** Differences between different cytokine levels in healing versus non-healing VLU (mean, standard deviation) [46,57].

Cytokines	Non-Healing VLU (pg/mL)	Healing VLU (pg/mL)
IL-1a	4610 (3194, 6027)	2355 (1782, 2928)
IL-1ra	7328 (6232, 8423)	5250 (4388, 6113)
IL-2	4.6 (2.2, 7.1)	2.4 (1.7, 3.1)
IL-3	0.58 (0.27, 0.89)	0.3 (0.09, 0.50)
IL-6	3550.5 (3258.4–3842.7)	3962.7 (3702–4223.3)
IL-9	1.9 (1.4, 2.3)	0.93 (0.66, 1.2)

**Table 6:** Comparison of acute wound fluid and chronic wound fluid in relation to healthy human serum [20,45,61,62,63].

Biochemical Component	Healing Exudate	Non-Healing Exudate
Serum components, i.e., Na^+^, K^+^, Cl^−^	Present	Present
Bicarbonate	Present	Lower than AWF and serum
Glucose	Lower than serum values	Lower than AWF and serum
Total protein	44 g/L (Lower than serum values of 73 g/L)	30 g/L Lower than AWF and serum
Albumin	25 g/L (Lower than serum values of 73 g/L)	17 g/L Lower than AWF and serum
C-reactive protein	Same as serum values	Raised levels—denotes inflammatory phase
Cytokines	Present	Increased levels (in particular IL-6 and TNF-a)
Matrix metalloproteinases	Present	Increased levels (in particular MMP-2 and MMP-9)
Tissue inhibitors of metalloproteinases	Present	Absent or low levels
Growth factors	Present	Degraded or completely absent
Vitronectin and Fibronectin	Present in their usual forms	Degraded into lower molecular-weight proteins
Free radicals	Low levels	Raised levels

**Table 7:** Comparison of healing wound fluid and non-healing exudate from VLU (median, range) [44].

Biochemical Component	Healing Exudate	Non-Healing Exudate
Bicarbonate (mmol/L)	19 (16–22)	17.5 (14–20)
Glucose (mmol/L)	2 (1.1–5.9)	1.2 (0.6–3.7)
Total protein (g/L)	41 (36–51)	34 (26–46)
Albumin (g/L)	23 (18–28)	19 (14–24)
C-reactive protein (mg/L)	5 (2.5–21)	13 (5–25)
Gamma globulin (g/L)	6 (4.4–9.0)	4.5 (3.9–6.6)
Cholesterol (mmol/L)	1.8 (1.3–3.2)	1.6 (1.2–3.2)

**Table 8:** Main differences in VLU exudate in VLU stages: infected, chronic, and healing.

Exudate Characteristic	Infected VLU	Chronic VLU	Healing VLU	References
**Volume**	>10 mL fluid/24 h	5 to 10 mL fluid/24 h	<5 mL fluid/24 h	[77,78]
**Type**	Purulent exudate	Serous to serosanguineous	Serous to absent	[29,79]

## Data Availability

Not applicable.
